# Supplementary material for: Reconciling Mining with the Conservation of Cave Biodiversity: A Quantitative Baseline to Help Establish Conservation Priorities
Source: PLoS One. 2016 Dec 20;11(12):e0168348. doi: 10.1371/journal.pone.0168348 (PMC5173368; doi:10.1371/journal.pone.0168348)
Supplement: S1 Dataset — (ZIP) [file pone.0168348.s002.zip › Taxa/Serra Norte/SN_2013/N5S/Lista N5S-57.pdf]

CAVIDADE N5S-0057

Classe	Ordem	Fam/Outros	Gên/Outros	Espécie	S-U	U-S	Zon.
Annelida	Oligochaeta			jovem		2	P
Arachnida	Acari	Anystidae	*Erythracarus*	sp.1		X	E/P
Arachnida	Acari	Cunaxidae		sp.1		X	E
Arachnida	Acari	Halacaridae		sp.1		X	E
Arachnida	Acari	Podocinidae	*Podocinum*	sp.1	X		E
Arachnida	Acari	Rhagidiidae		sp.4		X	E
Arachnida	Amblypygi	Charinidae	*Charinus*	sp.1	1	3	E/P
Arachnida	Amblypygi	Phrynidae	*Heterophrynus*	*longicornis*	1	2	E/P
Arachnida	Araneae	Araneidae	*Alpaida*	aff. *negro*		X	E
Arachnida	Araneae	Ctenidae		jovem		1	E
Arachnida	Araneae	Drymusidae	*Drymusa*	*spelunca*	X	X	P
Arachnida	Araneae	Ochyroceratidae	*Ochyrocera*	sp.1	X	X	E/P
Arachnida	Araneae	Pholcidae	*Mesabolivar*	*aurantiacus*	X	X	E
Arachnida	Araneae	Pholcidae	*Mesabolivar*	sp.1		X	
Arachnida	Araneae	Pisauridae		jovem		3	E/P
Arachnida	Araneae	Prodidomidae		jovem	X		E
Arachnida	Araneae	Scytodidae	*Scytodes*	jovem		1	E
Arachnida	Araneae	Theraphosidae	aff. *Holothele*	sp.1		1	E
Arachnida	Araneae	Theridiidae	*Theridion*	sp.1	X		E
Arachnida	Araneae	Theridiidae	*Theridion*	sp.4	X		P
Arachnida	Araneae	Theridiosomatidae	*Plato*	sp.1	X	X	E/P
Arachnida	Opiliones	Escadabiidae		sp.2		X	E
Arachnida	Opiliones	Sclerosomatidae	*Prionostema*	sp.1		1	E
Arachnida	Pseudoscorpiones	Chthoniidae		jovem	X		P
Chilopoda	Geophilomorpha	Schendylidae		sp.1	1		P
Diplopoda	Polydesmida	Pyrgodesmidae		sp.1	X	X	P
Entognatha	Collembola	Paronellidae		sp.1		X	E
Entognatha	Collembola	Paronellidae		sp.4	X		P
Entognatha	Collembola	Paronellidae		sp.6	X		E
Entognatha	Collembola	Sminthuroidea		sp.2		X	P
Insecta	Blattodea	Blattellidae		sp.1		1	E
Insecta	Coleoptera	Carabidae		sp.3	X		P
Insecta	Coleoptera	Hydrophilidae		sp.2	X		P
Insecta	Coleoptera	Staphylinidae		sp.32	X		P
Insecta	Coleoptera	Tenebrionidae		sp.1	X		P
Insecta	Coleoptera			jovem		X	E
Insecta	Diptera	Cecidomyiidae	Cecidomyiinae	sp.		X	E
Insecta	Diptera	Chironomidae		sp.	X		P
Insecta	Diptera	Phoridae	Metopininae	sp.	X		E
Insecta	Diptera	Psychodidae	Phlebotominae	sp.	X	X	E/P
Insecta	Diptera	Sciaridae		sp.	X		P
Insecta	Heteroptera	Veliidae	*Paravelia*	sp.	X	X	E/P
Insecta	Hymenoptera	Diapriidae		sp.2		X	E
Insecta	Hymenoptera	Formicidae	*Camponotus*	sp.1	X		E
Insecta	Hymenoptera	Formicidae	*Crematogaster*	sp.1	X		E
Insecta	Hymenoptera	Formicidae	*Paratrechina*	sp.1	X	X	E
Insecta	Hymenoptera	Formicidae	*Pheidole*	sp.4	X		P
Insecta	Hymenoptera	Formicidae	*Solenopsis*	sp.2		X	E
Insecta	Isoptera	Termitidae	*Nasutitermes*	sp.	X	X	E/P

Insecta	Lepidoptera			jovem	X		E/P
Insecta	Orthoptera	Phalangopsidae	*Paraclodes*	sp.	6	2	E/P
Insecta	Orthoptera	Phalangopsidae	*Phalangopsis*	sp.	2	40	E/P
Insecta	Plecoptera	Gripopterygidae		jovem		1	E
Insecta	Thysanura	Nicoletiidae		sp.1	X	X	E/P
Malacostraca	Isopoda	Armadillidae		sp.1	X		E
Malacostraca	Isopoda	Calabozoidea		sp.1	X		E
Turbellaria				sp.4	X	X	E/P
Amphibia	Anura	Strabomantidae	*Pristimantis*	cf. *fenestratus*	1		E
Mammalia	Chiroptera	Emballonuridae	*Peropteryx*	*kappleri*	1		E
Mammalia	Chiroptera	Furipteridae	*Furipterus*	*horrens*	3		E
